# Impact of the World Inflammatory Bowel Disease Day and Crohn’s and Colitis Awareness Week on Population Interest Between 2016 and 2020: Google Trends Analysis

**DOI:** 10.2196/32856

**Published:** 2021-10-28

**Authors:** Krixie Silangcruz, Yoshito Nishimura, Torrey Czech, Nobuhiko Kimura, Hideharu Hagiya, Toshihiro Koyama, Fumio Otsuka

**Affiliations:** 1 University of Hawaii Honolulu, HI United States; 2 Okayama University Okayama Japan

**Keywords:** inflammatory bowel disease, ulcerative colitis, Crohn disease, google trends, trend analysis, online health information, awareness, chronic disease, gastrointestinal, trend, impact, public health, United States

## Abstract

**Background:**

More than 6 million people are affected by inflammatory bowel disease (IBD) globally. The World IBD Day (WID, May 19) and Crohn’s and Colitis Awareness Week (CCAW, December 1-7) occur yearly as national health observances to raise public awareness of IBD, but their effects are unclear.

**Objective:**

The aim of this study was to analyze the relationship between WID or CCAW and the public health awareness on IBD represented by the Google search engine query data.

**Methods:**

This study evaluates the impact of WID and CCAW on the public awareness of IBD in the United States and worldwide from 2016 to 2020 by using the relative search volume of “IBD,” “ulcerative colitis,” and “Crohn’s disease” in Google Trends. To identify significant time points of trend changes (joinpoints), we performed joinpoint regression analysis.

**Results:**

No joinpoints were noted around the time of WID or CCAW during the study period in the search results of the United States. Worldwide, joinpoints were noted around WID in 2020 with the search for “IBD” and around CCAW in 2017 and 2019 with the search for “ulcerative colitis.” However, the extents of trend changes were modest without statistically significant increases.

**Conclusions:**

These results posed a question that WID and CCAW might not have worked as expected to raise public awareness of IBD. Additional studies are needed to precisely estimate the impact of health observances to raise the awareness of IBD.

## Introduction

Inflammatory bowel disease (IBD) is a global disease with an increasing prevalence in newly industrialized countries, and rising cases have been documented in every continent [1]. Recent systematic reviews have demonstrated that IBD is increasing in such countries [2]. Globally in 2017, there were 6.8 million cases of IBD with an increased age-standardized prevalence rate from 1990 to 2017 [2]. Within the United States, it was estimated that more than a million adult Americans had IBD [3,4].

Research into IBD, however, is largely underrepresented despite its prevalence owing to the multifactorial nature of the disease [5]. Global efforts have been made to raise the awareness of IBD. In 2010, the World IBD Day (WID) was created by the European Federation of Crohn’s and Ulcerative Colitis Association and patient organizations to increase IBD awareness and to provide education about IBD to the public [6,7]. Similarly, Crohn’s and Colitis Awareness Week (CCAW) was created by a US Senate resolution in 2011, with goals of encouraging all people in the United States to engage in activities aimed at raising awareness of IBD among the general public [8].

Disease awareness and health promotion campaigns are created to increase public health education, and awareness, and ultimately change behavior [9]. Approximately 200 health awareness days, weeks, or months are on the US National Health Observances calendar [10], and nearly 70% of these health awareness occasions have been introduced after 2005. Despite the increasing number of awareness initiatives, there is a lack of data regarding evidence of their effectiveness and impact [11]. This lack of data highlights the need for greater evaluation and quantifiable metrics to determine the impact of health behaviors on a global scale.

Because web-based searches are a predominant source of access to health awareness–related information, internet searches are a reflection of engagement between the public and resources, which increase disease awareness. Searches are individual proxies for public disease awareness and provide insight into the effect of dissemination of information via global public health days and weeks. Google Trends (GT) is a novel, open-source, freely accessible resource that allows researchers to analyze Google search query data [12]. An analysis regarding the efficacy and public health behaviors that resulted from IBD awareness initiatives has not been done previously. We aimed to perform a hypothesis generation if the WID or CCAW effectively increased the public health awareness for IBD through GT data by using joinpoint trend analysis.

## Methods

### Data Source

GT is a data source generated from the total Google search data [13]. These data are available to the public, and GT has been used in multiple social, public health, or global health research to dig into the public attention [14-25]. Surrogate of the public attention in GT is the relative popularity of specific search terms or topics in a certain category (eg, health), place, and time range. The relative popularity is defined as a relative search volume (RSV) with a scale of 0-100 (0 being the lowest popularity) [14,19-21]. The RSV correlates with how popular the terms are at a certain time point.

### Search Input

We followed protocols noted by previous studies [17,19,21]. Briefly, we accessed data between July 11 and 13, 2021, and chose [Inflammatory bowel disease], [Ulcerative colitis], and [Crohn’s disease] as search inputs. The location of the search included United States and worldwide.

### Search Variables

To specifically obtain the popularity of the disease-related search inputs, all searches were done with a “disease” option in the Health category (with a “disease” option, search volumes of subtopics or relevant themes are included). We chose each full year from 2016 to 2020 as search scales to visualize weekly trends of the RSVs (each year contains 52 or 53 weeks; the WID occurred in the 20th week of 2016-2019 and in the 19th week of 2020; CCAW occurred in the 48th-49th week in 2016-2019 and in the 49th-50th week in 2020).

### Statistical Analyses

We used a joinpoint regression model with the Joinpoint Regression Program version 4.9.0.0, March 2021 [26] to analyze the RSV data and their time trend. This software enables us to identify time points called joinpoints, where a temporal trend significantly changes. We defined the analysis criteria to look for up to 3 joinpoints. The weekly percentage changes between trend change points were determined with 95% CIs. The threshold for statistical significance was defined as a *P* value <.05, suggesting the level at which the slope differed from zero.

### Ethical Considerations

The publicly available data published by GT are utilized in the project [13]. This study was approved by the institutional review board of the Okayama University Hospital with a waiver for informed consent since the study intended to retrospectively analyze open data (1910-009). All research methods were performed in accordance with relevant guidelines and regulations.

## Results

### Trends in the Search Volume of Inflammatory Bowel Disease

**Figure 1:**
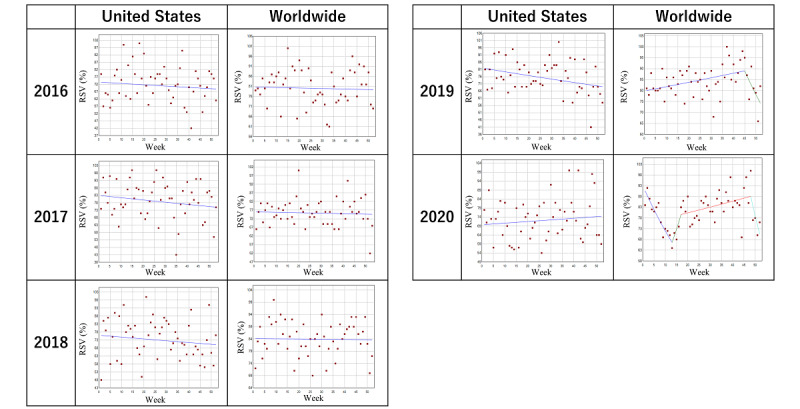
Trends in the relative search volume of inflammatory bowel disease during 2016-2020. Weekly relative search volumes for the search term “inflammatory bowel disease” are described. World Inflammatory Bowel Disease day occurred in the 20th week of 2016-2019 and the 19th week of 2020; Crohn’s & Colitis Awareness Week occurred in the 48th to 49th week in 2016-2019 and the 49th to 50th week in 2020. The number of slopes is determined by the number of joinpoints identified by the analysis. Joinpoints are the time points when statistically significant changes in the linear slopes are noted. RSV: relative search volume.

Table 1 and Figure 1 describe the trends and trend changes of the weekly RSVs for “inflammatory bowel disease” in each full year from 2016 to 2020. With respect to the search results in the United States, no joinpoints were observed throughout the period. Regarding the search results worldwide, there was a joinpoint at the 45th week in 2019 before which a significant increase in the weekly percentage change of 0.2% (95% CI 0.1-0.4) was observed. In 2020, a joinpoint was noted in the 17th week (3 weeks before WID), after which there was a significant weekly increase in the RSV by 0.3% (*P*<.001). Further, the third joinpoint was observed in the 48th week (a week prior to CCAW). However, no joinpoints were noted from 2015 to 2018 around the time of WID or CCAW.

**Table 1:** Trend changes in the relative search volumes of inflammatory bowel disease in 2016-2020.^a^

Country, year	Period 1		Period 2		Period 3		Period 4	
	Weeks	Weekly percentage change (%) (95% CI)	Weeks	Weekly percentage change (%) (95% CI)	Weeks	Weekly percentage change (%) (95% CI)	Weeks	Weekly percentage change (%) (95% CI)
United States, 2016	1-52	–0.1 (–0.4 to 0.2)	N/A^b^	N/A	N/A	N/A	N/A	N/A
United States, 2017	1-53	–0.2 (–0.5 to 0.1)	N/A	N/A	N/A	N/A	N/A	N/A
United States, 2018	1-52	–0.2 (–0.5 to 0.2)	N/A	N/A	N/A	N/A	N/A	N/A
United States, 2019	1-52	–0.3^c^ (–0.6 to 0)	N/A	N/A	N/A	N/A	N/A	N/A
United States, 2020	1-52	0.1 (–0.2 to 0.4)	N/A	N/A	N/A	N/A	N/A	N/A
Worldwide, 2016	1-52	0 (–0.2 to 0.2)	N/A	N/A	N/A	N/A	N/A	N/A
Worldwide, 2017	1-53	0 (–0.2 to 0.1)	N/A	N/A	N/A	N/A	N/A	N/A
Worldwide, 2018	1-52	0 (–0.2 to 0.2)	N/A	N/A	N/A	N/A	N/A	N/A
Worldwide, 2019	1-45	0.2^c^ (0.1 to 0.4)	45-52	–2.6 (–5.4 to 0.3)	N/A	N/A	N/A	N/A
Worldwide, 2020	1-13	–2.5^c^ (–3.6 to –1.4)	13-17	4.6 (–4.9 to 15.0)	17-48	0.3^c^ (0 to 0.6)	48-52	–5.4 (–10.9 to 0.5)

^a^Periods were separated as Period 1-4, when the trend changes were statistically detected in the joinpoint regression analysis during the study period.

^b^N/A: not applicable.

^c^Significantly different from zero (*P*<.05).

### Trends in the Search Volume of Ulcerative Colitis

**Figure 2:**
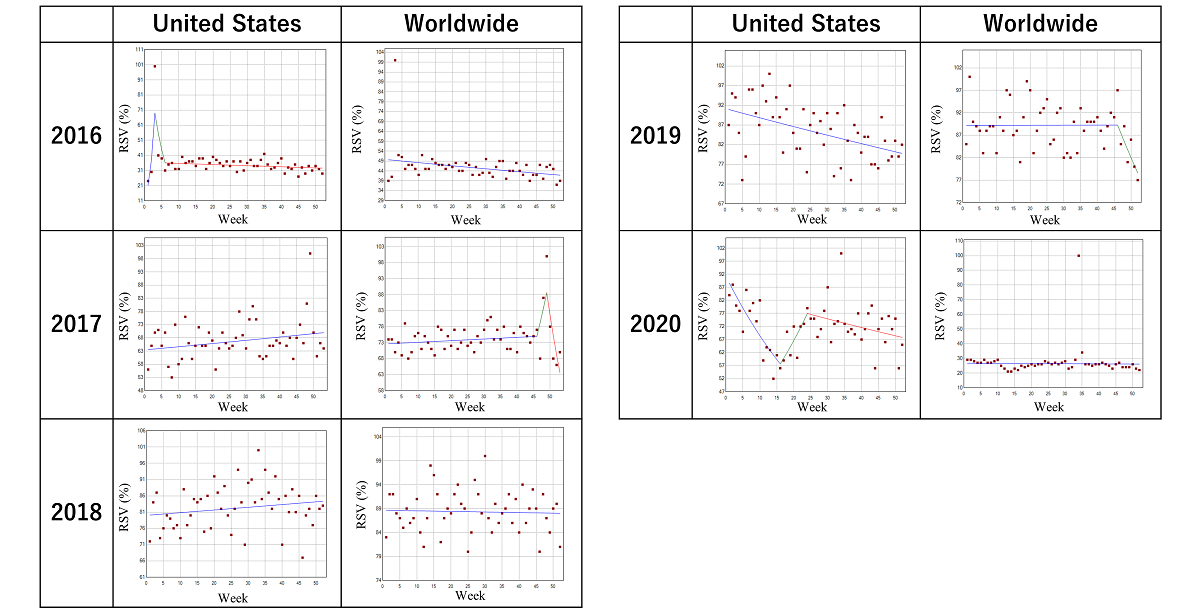
Trends in the relative search volume of ulcerative colitis during 2016-2020. Weekly relative search volumes for the search term “ulcerative colitis.” Except for the 16th week (4 weeks before World Inflammatory Bowel Disease Day) in the United States and the 49th week (Crohn’s and Colitis Awareness Week) worldwide in 2020, no other joinpoints were noted around the time of World Inflammatory Bowel Disease Day or Crohn’s and Colitis Awareness Week during the designated period. RSV: relative search volume.

Table 2 and Figure 2 describe the trends and trend changes of the weekly RSVs for ulcerative colitis in the designated period. In the search results of the United States and worldwide, a big surge was observed in the 3rd week in 2016. In 2020, a joinpoint was noted in the 16th week (4 weeks before WID), after which a nonstatistically significant but considerable weekly RSV increase by 3.7% (*P*<.001) was observed until the 24th week. For worldwide results, there was a prominent joinpoint in the 49th week (CCAW) in 2017. No other joinpoints were observed around the time of WID or CCAW in 2016 or 2018 to 2020.

**Table 2:** Trend changes in the relative search volumes of ulcerative colitis in 2016-2020.^a^

Country, year	Period 1		Period 2		Period 3	
	Weeks	Weekly percentage change (%) (95% CI)	Weeks	Weekly percentage change (%) (95% CI)	Weeks	Weekly percentage change (%) (95% CI)
United States, 2016	1-3	80.2^b^ (40.1 to 131.8)	3-6	–19.5 (–37.4 to 3.6)	6-52	–0.2^b^ (–0.4 to 0)
United States, 2017	1-53	0.2 (0 to 0.4)	N/A^c^	N/A	N/A	N/A
United States, 2018	1-52	0.1 (–0.1 to 0.3)	N/A	N/A	N/A	N/A
United States, 2019	1-52	–0.3^b^ (–0.4 to –0.1)	N/A	N/A	N/A	N/A
United States, 2020	1-16	–2.8^b^ (–4.0 to –1.6)	16-24	3.7 (–0.3 to 7.8)	24-52	–0.5 (–0.9 to 0)
Worldwide, 2016	1-52	–0.3^b^ (–0.6 to –0.1)	N/A	N/A	N/A	N/A
Worldwide, 2017	1-46	0.1 (0 to 0.2)	46-49	5.7 (–6.5 to 19.6)	49-53	–7.9^b^ (–11.5 to –4.3)
Worldwide, 2018	1-52	0 (–0.1 to 0.1)	N/A	N/A	N/A	N/A
Worldwide, 2019	1-46	0 (–0.1 to 0.1)	46-52	–2.1 (–4.5 to 0.3)	N/A	N/A
Worldwide, 2020	1-52	0 (–0.4 to 0.3)	N/A	N/A	N/A	N/A

^a^Periods were separated as Period 1-4, when the trend changes were statistically detected in the joinpoint regression analysis during the study period.

^b^Significantly different from zero (*P*<.05).

^c^N/A: not applicable.

### Trends in the Search Volume of Crohn disease

**Figure 3:**
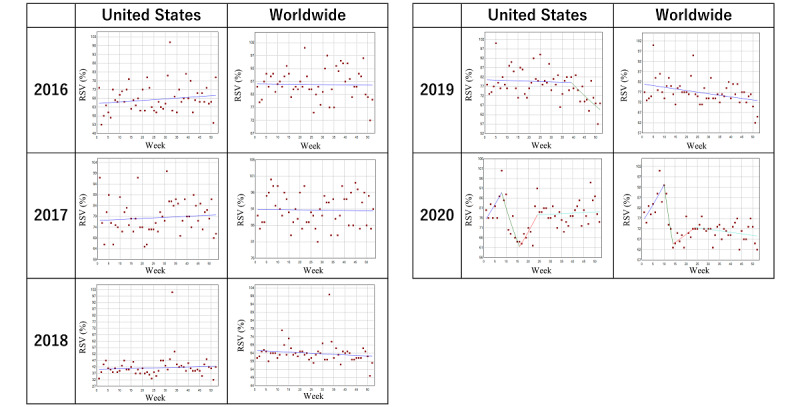
Trends in the relative search volume of Crohn disease during 2016-2020. Weekly relative search volumes for the search term “Crohn’s disease.” No joinpoints were noted around the time of World Inflammatory Bowel Disease Day or Crohn’s and Colitis Awareness Week throughout the period. RSV: relative search volume.

Table 3 and Figure 3 describe the trends and trend changes in the weekly RSVs for Crohn disease in the designated period. Between 2017 and 2019, there was no remarkable trend change in both the United States and worldwide. In 2020, joinpoints were observed in the 8th week, the 16th week, and the 24th week in the United States. For worldwide, joinpoints were observed in the 10th week, the 14th week, and the 24th week. However, there were no joinpoints around the time of WID or CCAW throughout the period.

**Table 3:** Trend changes in the relative search volumes of Crohn disease during 2016-2020.^a^

Country, year	Period 1		Period 2		Period 3		Period 4	
	Weeks	Weekly percentage change (%) (95% CI)	Weeks	Weekly percentage change (%) (95% CI)	Weeks	Weekly percentage change (%) (95% CI)	Weeks	Weekly percentage change (%) (95% CI)
United States, 2016	1-52	0.1 (–0.1 to 0.3)	N/A^b^	N/A	N/A	N/A	N/A	N/A
United States, 2017	1-53	0.1 (–0.1 to 0.2)	N/A	N/A	N/A	N/A	N/A	N/A
United States, 2018	1-52	0.1 (–0.2 to 0.4)	N/A	N/A	N/A	N/A	N/A	N/A
United States, 2019	1-39	0 (–0.3 to 0.2)	39-52	–1.5^c^ (–2.8 to –0.3)	N/A	N/A	N/A	N/A
United States, 2020	1-8	2.3 (–0.4 to 5.1)	8-16	–4.5^c^ (–7.0 to –1.9)	16-24	2.9^c^ (0.2 to 5.7)	24-52	0.1 (–0.3 to 0.4)
Worldwide, 2016	1-52	0 (–0.1 to 0.1)	N/A	N/A	N/A	N/A	N/A	N/A
Worldwide, 2017	1-53	0 (–0.1 to 0.1)	N/A	N/A	N/A	N/A	N/A	N/A
Worldwide, 2018	1-52	–0.1 (–0.3 to 0.1)	N/A	N/A	N/A	N/A	N/A	N/A
Worldwide, 2019	1-52	–0.2 (–0.3 to −0.1)	N/A	N/A	N/A	N/A	N/A	N/A
Worldwide, 2020	1-10	2.2^c^ (0.6 to 3.8)	10-14	–8.7^c^ (–16.4 to –0.4)	14-24	1.2 (–0.4 to 2.8)	24-52	–0.2 (–0.5 to 0.1)

^a^Periods were separated as Period 1-4, when the trend changes were statistically detected in the joinpoint regression analysis during the study period.

^b^N/A: not applicable.

^c^Significantly different from zero (*P*<.05).

## Discussion

This study evaluated how the global campaigns for promoting IBD, such as WID and CCAW, affected public awareness by using the RSVs of GT data as a surrogate. Although there were several significant joinpoints for IBD, Crohn disease, and ulcerative colitis, overall, the results in this study posed a hypothesis that WID and CCAW might not have affected the public interest in the United States and worldwide. Rather, the RSVs seem to have been affected by timely topics. For example, in the 3rd week of 2016, when there was a significant increase in the RSV of ulcerative colitis in the United States, a famous US singer-songwriter reportedly passed away due to the disease. In March 2020, when significant trend changes were observed in the United States and worldwide, a well-known American comedian revealed that he had Crohn disease. Similarly, when the same comedian was featured in a film about a man who has Crohn disease in June 2020, there were trend changes both in the United States and worldwide (24th week). Only in 2017 worldwide, considerable trend changes in the RSVs for ulcerative colitis were noted around CCAW, although the weekly percentage change was not statistically significant. Given the rapid increase in the global prevalence of IBD with increasing health care costs, raising public awareness of IBD is a pressing global health issue. While one would think that people may be more aware of IBD, given the rising number of IBD cases worldwide, more efforts are needed to rigorously evaluate if public awareness of IBD has trended up or not.

Since 2020, the dramatic challenges of the COVID-19 pandemic have greatly affected our lives, which might have affected the public interests for IBD as well. Because immunocompromised patients may be vulnerable to COVID-19, there were concerns about whether patients with IBD might be more susceptible to COVID-19 and have worser outcomes [27]. In a cross-sectional questionnaire, patients with IBD were apprehensive about the COVID-19 pandemic, as they felt more vulnerable to COVID-19 owing to their condition and their immunosuppressive therapies, including biologics. Many patients also felt disturbed, depressed, and tense when thinking of the infection [28]. To provide solid supports for patients with IBD during the pandemic, further efforts to increase public awareness of the entity are crucial. One example of a successful public health awareness campaign is the annual breast cancer awareness campaign [29], which achieved appropriate identification of targets, early involvement of the key stakeholders such as celebrities with the condition, and utilization of smartphone apps or eHealth platforms even during the current pandemic.

This study's strength is that this is the first hypothesis-generating study to see the extent of public awareness of IBD in the United States and worldwide by using the GT database. Using the open data, we could quantify the current trends of general interest in IBD. However, several limitations need to be addressed. First, owing to the nature of GT, the results of this study only included results from those who had internet access and sought health-related information via Google search. Given the high internet penetration rates—approximately 90.4% in North America and 60.1% worldwide [30]—and high US Google search market share of approximately 83% [31], GT is considered a good surrogate of public awareness. Second, GTs are proxies for engagement. Sentinel surveillance such as surveys may be needed to clarify the findings. Third, the potential effect on RSVs may lag the intervention by weeks, and it is uncertain how long the effects of the intervention would last, making it challenging to assess the impact of the intervention as RSVs. Fourth, there are confounders such as separate media coverage of the disease, which are difficult to identify and account for the uncertainty about how to attribute to the independent variable of interest. Further, incorporating the analysis of Google search query data of the actual public awareness campaigns (in this case, WID and CCAW) might be a preferred approach to reduce confounding factors and directly evaluate the effects of these health observances to garner an audience. However, the RSVs for WID and CCAW were too few to conduct a joinpoint analysis during the study period (Multimedia Appendix 1). No joinpoints were noted around the time of WID or CCAW throughout the period. Regarding WID, there were spikes in the RSV in the week of WID on May 19 (worldwide search in 2016 to 2020 and the US search in 2018 and 2020). Otherwise, the RSVs were consistently zero. For CCAW, GT could not return queries since there were too few Google searches using the term. Despite these limitations, our approach is interestingly novel to generate hypotheses on campaign effectiveness or ineffectiveness in the public awareness of IBD.

In conclusion, using the GT data as a surrogate, our study posed a possibility that WID and CCAW might not have successfully improved public awareness toward IBD. There is a need to look deeper into how to precisely assess the public awareness and improve public awareness using these health observances based on good examples.

## Appendix

Multimedia Appendix 1 Weekly relative search volumes of World Inflammatory Bowel Disease Day and Crohn’s and Colitis Awareness Week in 2016-2020.

## Abbreviations

CCAW: Crohn’s and Colitis Awareness Week
GT: Google trends
IBD: inflammatory bowel disease
RSV: relative search volume
WID: World IBD Day
