# Supplementary figures and images for: Impact of the World Inflammatory Bowel Disease Day and Crohn’s and Colitis Awareness Week on Population Interest Between 2016 and 2020: Google Trends Analysis
Source: JMIR Infodemiology. 2021 Oct 28;1(1):e32856. doi: 10.2196/32856 (PMC9987196; doi:10.2196/32856)

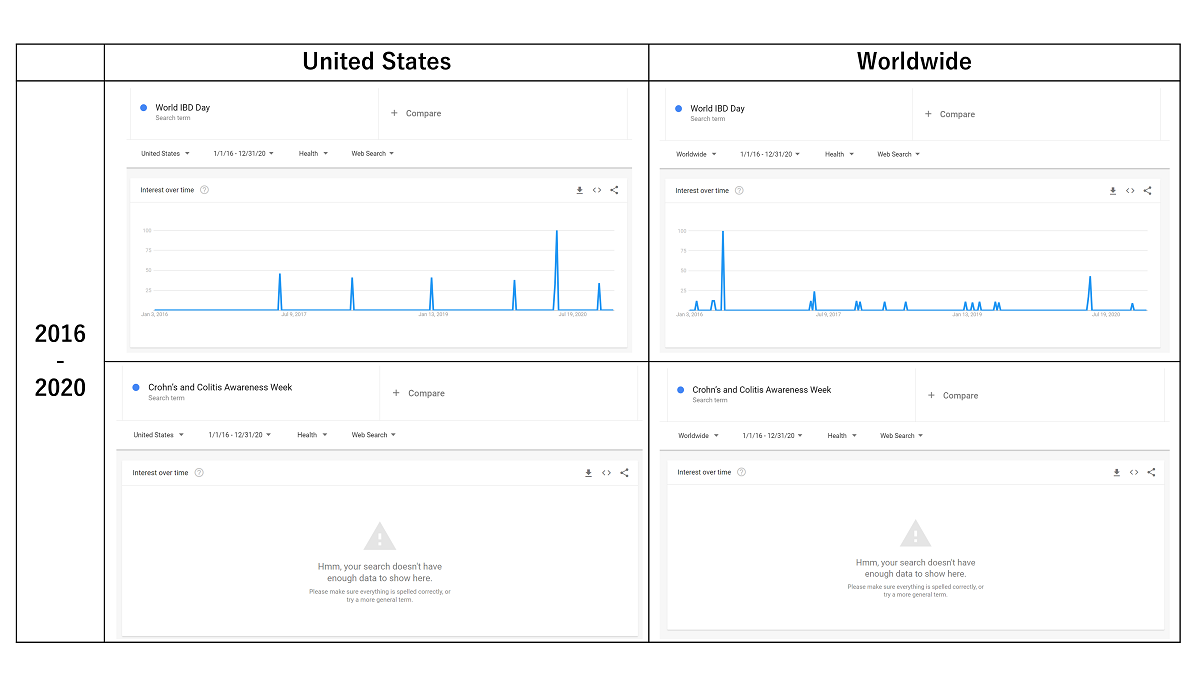

Supplement: Multimedia Appendix 1 [file infodemiology_v1i1e32856_app1.png]
